# Third-Trimester HIV Viremia Treated With Long-Acting Cabotegravir and Rilpivirine: A Case Report

**DOI:** 10.1093/ofid/ofaf604

**Published:** 2025-10-01

**Authors:** Noora Kazanji, Blake Max, Rodrigo M Burgos, Jennifer T Pham, Catherine E Ford

**Affiliations:** Division of Infectious Diseases, University of Illinois at Chicago, Chicago, Illinois, USA; Department of Pharmacy Practice, University of Illinois at Chicago, Chicago, Illinois, USA; Division of Infectious Diseases, University of Illinois at Chicago, Chicago, Illinois, USA; Department of Pharmacy Practice, University of Illinois at Chicago, Chicago, Illinois, USA; Department of Pharmacy Practice, University of Illinois at Chicago, Chicago, Illinois, USA; Department of Pediatrics, Division of Neonatology, University of Illinois at Chicago, Chicago, Illinois, USA; Department of Obstetrics and Gynecology, University of Illinois at Chicago, Chicago, Illinois, USA

**Keywords:** CAB, HIV, LA, viremia pregnancy

## Abstract

We report the first case to quantitatively demonstrate a rapid and substantial viral load reduction using long-acting cabotegravir and rilpivirine in the third trimester of pregnancy. This case underscores the challenges some pregnant individuals face with daily oral antiretroviral therapy and the potential role of long-acting injectables in select scenarios.

## CASE REPORT

A 31-year-old gravida 4 para 3 woman was newly diagnosed with human immunodeficiency virus (HIV) type 1 at 9 weeks’ gestation during her initial prenatal visit. Her obstetric history included 3 cesarean deliveries, all performed over 3 years prior to this pregnancy. She had no significant medical comorbidities and was taking only a prenatal vitamin. Her body mass index (BMI) was 18.1 kg/m².

Due to transportation and childcare barriers, she was lost to follow-up and did not initiate antiretroviral therapy (ART) with bictegravir/tenofovir alafenamide/emtricitabine (BIC/TAF/FTC) until 22 weeks’ gestation. At that time, her HIV-1 RNA was 14 600 copies/mL and her absolute CD4 count was 686 cells/µL (38%). Genotypic resistance testing via next-generation sequencing (NGS) identified a K103N mutation; no resistance-associated mutations (RAMs) or accessory mutations affecting cabotegravir (CAB) or rilpivirine (RPV) were detected (see Supplementary Table 1).

She reported long-standing pill aversion dating back to childhood, though she denied dysphagia or odynophagia. Several strategies were discussed to facilitate adherence, including masking taste with peanut butter or acidic beverages. She subsequently missed her follow-up appointment and was not seen again until 29 weeks’ gestation, at which point her viral load (VL) had increased to 19 800 copies/mL. She acknowledged taking approximately 3 doses of BIC/TAF/FTC per week.

At 31 weeks’ gestation, her VL had declined to 7600 copies/mL. Given continued suboptimal adherence, she was transitioned to a crushed formulation of abacavir/lamivudine/dolutegravir (ABC/3TC/DTG), administered once daily mixed with applesauce or pudding. At 35 weeks, her HIV-1 RNA had increased to 9900 copies/mL. She reported consistent daily adherence and denied loss of drug during preparation. Repeat NGS testing again identified the K103N mutation without additional resistance mutations. She remained clinically stable with no reported symptoms, such as vaginal bleeding, contractions, or fluid leakage, and reported normal fetal movements.

**Figure 1.**
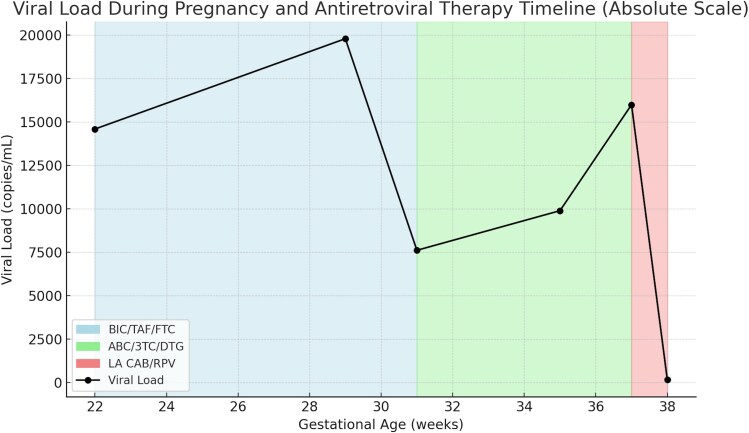
Patient's VL during pregnancy while on different ART regimens.

At 37 weeks’ gestation, she received her first dose of long-acting intramuscular CAB and RPV (LA CAB/RPV), which was well tolerated. Her HIV-1 RNA on the day of injection was 16 600 copies/mL. At 38 weeks, she was admitted for a scheduled repeat low-transverse cesarean delivery due to viremia. On the morning of her surgery, an HIV-1 RNA was obtained at 08:30 which measured 171 copies/mL (Figure 1). At 09:12, she was initiated on intravenous zidovudine with a 2 mg/kg loading dose followed by a 1 mg/kg/h infusion. She then underwent cesarean delivery, which was complicated by dense fascial adhesions from prior surgeries; however, intra-abdominal adhesions were minimal. The procedure was completed without complication.

The newborn was initiated on oral zidovudine (4 mg/kg/dose BID), lamivudine (2 mg/kg/dose BID), and raltegravir (1.5 mg/kg/dose daily). Despite these interventions, HIV-1 infection was detected in the infant by nucleic acid testing on the first day of life and confirmed with subsequent testing 2 weeks later with an HIV-1 VL of 181 copies/mL. The infant's genotype was identical to the mother's, including the presence of K103N as the sole NNRTI RAM. No additional RAM's or minority variants were detected. Both mother and infant recovered well postoperatively. The mother received her second injection of LA CAB/RPV 1 month later, at which time her VL was undetectable. The infant's absolute CD4 was 2835 cells/µL (62%) at 1 month of life.

## DISCUSSION

This is the first reported case to quantitatively demonstrate a rapid and substantial VL reduction using LA CAB/RPV in the third trimester of pregnancy. In our patient, HIV-1 RNA declined from 16 600 copies/mL to 171 copies/mL within 1 week following a single dose, highlighting the potential role of LA CAB/RPV in late pregnancy when adherence to oral ART is compromised.

One of the benefits of integrase strand transfer inhibitors (INSTI) and why they are universally recommended on all HIV treatment guidelines is the rapid reduction of HIV-RNA. In a phase 3 trial, treatment-naïve participants randomized to ABC/3TC/DTG achieved suppression in a median of 28 days, compared to 84 days with efavirenz/tenofovir disoproxil/emtricitabine [1]. Oral CAB, which is an analog of DTG, demonstrated a mean reduction of 2.0 log₁₀ copies/mL in VL after 7 days of treatment in a preclinical dose-ranging study [2]. There have been few studies of viremic patients initiating LA CAB/RPV, but data on timing of HIV-RNA reduction are unknown.

Daily oral ART remains the standard of care for pregnant individuals with HIV, but barriers such as nausea, vomiting, pill aversion, psychosocial stressors, or trauma can impair adherence. Despite multiple trials of high-potency regimens—including BIC/TAF/FTC and crushed ABC/3TC/DTG—our patient was unable to achieve viral suppression due to persistent pill aversion. This case underscores a critical gap in treatment options for a subset of pregnant individuals who are unable to tolerate oral ART.

In cases like this, we are treating 2 patients—the pregnant individual and the fetus—whose health is directly tied to achieving maternal virologic suppression. While the substantial VL decline observed after 1 injection is encouraging, it remains unknown whether earlier administration of LA CAB/RPV could have prevented mother-to-child (MTC) perinatal transmission in this case. This uncertainty underscores the urgent need for robust data on the use of LA CAB/RPV in pregnancy to better equip clinicians to prevent such outcomes.

Current guidelines do not recommend LA CAB/RPV initiation during pregnancy due to limited pharmacokinetic (PK) and safety data. However, recent studies suggest promising outcomes. In a compassionate use program, 35 people with HIV (PWH)—including 28 with viremia (median VL 60 300 copies/mL) and 11 with perinatal HIV—received LA CAB/RPV. Among perinatally infected participants who continued therapy, 8 achieved virologic suppression; resistance outcomes for the remaining 3 were not reported [3]. A multicenter retrospective review was presented at the 2025 Long-Acting Anti-Infectives conference that described 31 pregnant individuals receiving LA CAB/RPV—23 initiated pre-pregnancy and 8 during gestation. All 8 initiated during pregnancy received monthly injections. At the initial prenatal visit, 6 had a VL greater than 200 copies/mL. At delivery, 25 had a maternal VL less than 20 copies/mL and 4 less than 200 copies/mL; 2 patients had a VL greater than 1000 copies/mL [4]. Although bimonthly LA CAB/RPV dosing has been associated with lower RPV concentrations in pregnancy, physiologic modeling studies suggest that both monthly and bimonthly regimens can maintain therapeutic levels, with monthly dosing offering more consistent exposure [5]. Additional safety data on CAB in pregnancy come from the HPTN 084 open-label extension, which found no difference in pregnancy or fetal outcomes among individuals who became pregnant while receiving CAB for pre-exposure prophylaxis and no change in PK half-life during pregnancy [6]. While this is a different clinical context, it supports the notion that RPV PK, which may be reduced during pregnancy, are more likely to drive concern around LA CAB/RPV use in this setting.

LA CAB/RPV is FDA-approved only for use in patients with viral suppression (HIV-1 RNA less than 50 copies/mL). Limited data suggest that LA CAB/RPV may be considered in select PWH who are unable to achieve or maintain suppression on oral ART despite intensive adherence support, contributing to recent updates in the International Antiviral Society–USA and US Department of Health and Human Services HIV treatment guidelines [7, 8]. Yet, resistance and efficacy remain a key concern when used off-label in viremic individuals. In an observational cohort from Ward 86 in San Francisco, 59 patients with baseline viremia (median VL 42 900 copies/mL) received LA CAB/RPV. At 48 weeks, 47 achieved viral suppression, but 5 developed virologic failure with resistance mutations to non-nucleoside reverse transcriptase inhibitors (NNRTIs) and/or INSTIs—3 of whom had received on-time injections [9, 10]. Further data from the D’Amico compassionate use series showed that 16 of 28 viremic patients (57%) achieved suppression, while 7 discontinued therapy due to incomplete virologic response. Six developed emergent NNRTI or INSTI resistance mutations not detected at baseline, including one with CAB resistance [1]. In the TRIO HIV Research Network cohort, 111 individuals with baseline viremia initiated LA CAB/RPV, including 54 with VL greater than 200 copies/mL and 57 with low-level viremia. Of the 81 with follow-up data, 89% achieved virologic suppression, with a higher proportion in the low-level viremia group (93% vs 84%). Two individuals experienced confirmed virologic failure (CVF) after initial suppression—one of whom developed RPV-RAM (L100I and K101E) [11].

We would also like to highlight the patient's baseline K103N mutation. While this mutation does not confer phenotypic resistance to RPV and was not exclusionary in the ATLAS, FLAIR, or ATLAS-2M trials [12–14], rare cases of CVF have been reported in individuals with baseline K103N receiving LA CAB/RPV. In a single site observational case series, 3 of 75 participants who were virologically suppressed at time of switch to LA CAB/RPV developed CVF, despite on-time injections. One individual had a baseline K103N without other NNRTI RAMs and later developed integrase resistance (N155H) [15]. However, in a pooled analysis by Orkin et al of 1651 participants across the phase 3 trials, 1.4% (*n* = 23) experienced CVF through 152 weeks, with no cases of CVF occurring among those with K103N alone (0/18), or among those with K103N plus other non-RPV NNRTI mutations (0/3) [16]. Factors significantly associated with increased CVF risk included the presence of RPV-RAMs, HIV-1 subtype A6/A1, and BMI ≥ 30 kg/m² [16]. In our patient's case, NGS identified K103N as the only NNRTI RAM, with no accessory resistance mutations. Although several reverse transcriptase polymorphisms were present, none are predicted to affect RPV or CAB susceptibility based on the Stanford HIV Drug Resistance Database [17]. While K103N may serve as a marker of prior NNRTI exposure and possible archived RPV resistance mutations below the detection threshold of conventional sequencing, our patient's rapid virologic response following LA CAB/RPV, despite the presence of K103N, supports clinical trial data that this mutation alone does not preclude treatment success and adds to the nuanced real-world evidence guiding selection for LA CAB/RPV.

Given that LA CAB/RPV was planned for postpartum use, we initially aimed to preserve its viability by avoiding premature use that might risk resistance. Therefore, we transitioned her to crushed ABC/3TC/DTG—a regimen with better-documented bioavailability and efficacy in achieving virologic suppression when crushed compared to BIC/TAF/FTC [18]. The shared decision to use LA CAB/RPV in our patient was made late in pregnancy, balancing the potential benefit of rapid VL reduction against the limited safety data and possible emergence of resistance. She tolerated the injection well and experienced a 2-log reduction in VL after 1 week. Given the rapid decrease in VL within 7 days just prior to delivery, this case highlights that 70% of the risk of HIV MTC perinatal transmission lies prior to delivery: 20% prior to week 36 and 50% between week 36 and prior to delivery [19].

## CONCLUSION

This is the first reported case to quantitatively demonstrate a substantial VL reduction using LA CAB/RPV in the third trimester of pregnancy after 1 week, following a single dose. This case highlights the challenges that some pregnant patients face in achieving virologic suppression on oral ART. Although LA CAB/RPV in any PWH with viremia may introduce RAM and virologic failure, it should be considered in select patients, particularly when all other treatment options have been exhausted and transmission is a concern. Further studies are urgently needed to evaluate the safety, pharmacokinetics, and efficacy of LA CAB/RPV during pregnancy to support its use in ultimately preventing perinatal HIV transmission.

## Supplementary Material

ofaf604_Supplementary_Data

## Supplementary Data

Supplementary materials are available at *Open Forum Infectious Diseases* online. Consisting of data provided by the authors to benefit the reader, the posted materials are not copyedited and are the sole responsibility of the authors, so questions or comments should be addressed to the corresponding author.

## References

[1] Walmsley SL, Antela A, Clumeck N, et al Dolutegravir plus abacavir-lamivudine for the treatment of HIV-1 infection. N Engl J Med 2013; 369:1807–18. 24195548 10.1056/NEJMoa1215541

[2] Spreen W, Min S, Ford SL, et al Pharmacokinetics, safety, and monotherapy antiviral activity of GSK1265744, an HIV integrase strand transfer inhibitor. HIV Clin Trials 2013; 14:192–203. 24144896 10.1310/hct1405-192

[3] D'Amico R, Cenoz Gomis S, Moodley R, et al Compassionate use of long-acting cabotegravir plus rilpivirine for people living with HIV-1 in need of parenteral antiretroviral therapy. HIV Med 2023; 24:202–11. 35945163 10.1111/hiv.13370

[4] Short RW, Zimmerman M, Aziz M, et al Long-acting cabotegravir/rilpivirine in pregnancy [oral presentation]. Presented at: 1st International Workshop on Long-Acting Anti-Infectives (LAAI); May 21–22, 2025; New Orleans, LA.

[5] van der Wekken-Pas L, Weiss F, Simon-Zuber C, et al Long-acting injectable cabotegravir and rilpivirine in a pregnant woman with HIV. Clin Infect Dis 2024; 79:1468–71. 38703388 10.1093/cid/ciae242 PMC11650856

[6] Delany-Moretlwe S, Hanscom B, Guo X, et al Evaluation of long-acting cabotegravir safety and pharmacokinetics in pregnant women in eastern and Southern Africa: a secondary analysis of HPTN 084. J Int AIDS Soc 2025; 28:e26401. 39748218 10.1002/jia2.26401 PMC11695207

[7] Sax PE, Thompson MA, Saag MS; IAS-USA Treatment guidelines panel. Updated treatment recommendation on use of cabotegravir and rilpivirine for people with HIV from the IAS-USA guidelines panel. JAMA 2024; 331:1060–1. 38427337 10.1001/jama.2024.2985

[8] U.S. Department of Health and Human Services . What's new in the guidelines. Clinicalinfo.HIV.gov. Updated Sep 12, 2024. Available at: https://clinicalinfo.hiv.gov/en/guidelines/hiv-clinical-guidelines-adult-and-adolescent-arv/whats-new

[9] Gandhi M, Hickey M, Imbert E, et al Demonstration project of long-acting antiretroviral therapy in a diverse population of people with HIV. Ann Intern Med 2023; 176:969–74. 37399555 10.7326/M23-0788 PMC10771861

[10] Hickey MD, Gistand N, Grochowski J, et al Viral suppression rates at 48 weeks in people with HIV starting long-acting cabotegravir/rilpivirine with initial viremia. Clin Infect Dis 2025; 80:864–70. 39367871 10.1093/cid/ciae500 PMC12043055

[11] Elion RA, Frick AJ, Radtchenko J, et al Utilization and effectiveness of cabotegravir plus rilpivirine in people with HIV (PWH) with viremia at treatment initiation [oral presentation]. Presented at: 1st International Workshop on Long-Acting Anti-Infectives (LAAI); May 21–22, 2025; New Orleans, LA.

[12] Swindells S, Andrade-Villanueva JF, Richmond GJ, et al Long-acting cabotegravir and rilpivirine for maintenance of HIV-1 suppression. N Engl J Med 2020; 382:1112–23. 32130809 10.1056/NEJMoa1904398

[13] Orkin C, Oka S, Philibert P, et al Long-acting cabotegravir plus rilpivirine for treatment in adults with HIV-1 infection: 96-week results of the randomised, open-label, phase 3 FLAIR study. Lancet HIV 2021; 8:e185–96. 33794181 10.1016/S2352-3018(20)30340-4

[14] Overton ET, Richmond G, Rizzardini G, et al Long-acting cabotegravir and rilpivirine dosed every 2 months in adults with human immunodeficiency virus 1 type 1 infection: 152-week results from ATLAS-2M, a randomized, open-label, phase 3b, noninferiority study. Clin Infect Dis 2023; 76:1646–54. 36660819 10.1093/cid/ciad020 PMC10156123

[15] Shankaran S, Hernandez-Guarin L, Jhobalia N, et al Virologic failure with cabotegravir-rilpivirine injections: a single site experience. Presented at: Conference for Retroviruses and Opportunistic Infections (CROI); March 3–6, 202; Denver, CO.

[16] Orkin C, Schapiro JM, Perno CF, et al Expanded multivariable models to assist patient selection for long-acting cabotegravir + rilpivirine treatment: clinical utility of a combination of patient, drug concentration, and viral factors associated with virologic failure. Clin Infect Dis 2023; 77:1423–31. 37340869 10.1093/cid/ciad370 PMC10654860

[17] Shafer RW . Rationale and uses of a public HIV drug-resistance database. J Infect Dis 2006; 194:S51–8. 16921473 10.1086/505356 PMC2614864

[18] Chrdle A, Jerhotová Z, Vacík M, Linka M, Chmelík V. Crushed dolutegravir/abacavir/lamivudine given via nasogastric tube in gastric outlet obstruction caused by cancer resulted in rapid viral load suppression. Int J STD AIDS 2018; 30:94–8. 30231834 10.1177/0956462418797847

[19] Kourtis AP, Bulterys M, Nesheim SR, Lee FK. Understanding the timing of HIV transmission from mother to infant. JAMA 2001; 285:709–12. 11176886 10.1001/jama.285.6.709

