# Supplementary material for: Third-Trimester HIV Viremia Treated With Long-Acting Cabotegravir and Rilpivirine: A Case Report
Source: Open Forum Infect Dis. 2025 Oct 1;12(10):ofaf604. doi: 10.1093/ofid/ofaf604 (PMC12547495; doi:10.1093/ofid/ofaf604)
Supplement: ofaf604_Supplementary_Data [file ofaf604_supplementary_data.docx]

**Supplementary Table S1**

Patient’s Baseline HIV-1 Genotype by Next Generation Sequencing

| HIV-1 Drug Resistance by NGS | See Note |
| --- | --- |
| Comment: Integrase Strand Transfer Inhibitor Drug Class           Bictegravir,BIC             Susceptible           Cabotegravir,CAB            Susceptible           Dolutegravir,DTG            Susceptible           Elvitegravir,EVG            Susceptible           Raltegravir,RAL             Susceptible             IN drug resistance mutations identified:  None             IN accessory resistance mutations identified:  None             IN additional mutations identified:  S17N, M50I, I72V, I84M, L101I, K111Q, T124A, T125A, K156R, I267V             IN uncalled sites identified:  None   Protease Inhibitor Drug Class           Atazanavir,ATV              Susceptible           Darunavir,DRV               Susceptible           Fosamprenavir,FPV           Susceptible           Indinavir,IDV               Susceptible           Lopinavir,LPV               Susceptible           Nelfinavir,NFV              Susceptible           Saquinavir,SQV              Susceptible           Tipranavir,TPV              Susceptible             PR drug resistance mutations identified:  None             PR accessory resistance mutations identified:  None             PR additional mutations identified:  T12A, M36I, L63P, I64V, H69Q, V77I, V82I, I93L             PR uncalled sites identified:  None   Nucleoside Reverse Transcriptase Inhibitor Drug Class           Abacavir,ABC                Susceptible           Zidovudine,AZT              Susceptible           Stavudine,D4T               Susceptible           Didanosine,DDI              Susceptible           Emtricitabine,FTC           Susceptible           Lamivudine,LMV              Susceptible           Tenofovir,TDF               Susceptible             NRTI drug resistance mutations identified:  None   Non-nucleoside Reverse Transcriptase Inhibitor Drug Class           Doravirine,DOR              Susceptible           Efavirenz,EFV               High-Level Resistance           Etravirine,ETR              Susceptible           Nevirapine,NVP              High-Level Resistance           Rilpivirine,RPV             Susceptible             NNRTI drug resistance mutations identified:  K103N             RT accessory resistance mutations identified:  None             RT additional mutations identified: V21I, V60I, K102R, K104R, K122E, K173N, Q174K, I178M, V179I, G196E, T200A, V245T, I293V, M357T, R358K, A376T, S379C, V381I, T386P, K390R, E399G, A400T, E404D, I434V, V435A             RT uncalled sites identified:  None   HIVGenotyper software version: 2.1.1.0 | |

Stanford HIV Drug Resistance Database Version: HIVDB_9.6
